# Comprehensive Analyses of miRNA-mRNA Network and Potential Drugs in Idiopathic Pulmonary Arterial Hypertension

**DOI:** 10.1155/2020/5156304

**Published:** 2020-07-03

**Authors:** Chan Li, Zeyu Zhang, Qian Xu, Ruizheng Shi

**Affiliations:** ^1^Department of Cardiovascular Medicine, Xiangya Hospital, Central South University, Changsha, Hunan, China; ^2^Department of Hepatobiliary Surgery, Xiangya Hospital, Central South University, Changsha, Hunan, China; ^3^Department of Cardiovascular Surgery, Xiangya Hospital, Central South University, Changsha, Hunan, China

## Abstract

**Introduction:**

Idiopathic pulmonary arterial hypertension (IPAH) is a severe cardiopulmonary disease with a relatively low survival rate. Moreover, the pathogenesis of IPAH has not been fully recognized. Thus, comprehensive analyses of miRNA-mRNA network and potential drugs in IPAH are urgent requirements.

**Methods:**

Microarray datasets of mRNA and microRNA (miRNA) in IPAH were searched and downloaded from Gene Expression Omnibus (GEO). Differentially expressed genes (DEGs) and differentially expressed miRNAs (DEMIs) were identified. Then, the DEMI-DEG network was conducted with associated comprehensive analyses including Gene Ontology (GO) analysis, Kyoto Encyclopedia of Genes and Genomes (KEGG) pathway enrichment analysis, and protein-protein interaction (PPI) network analysis, while potential drugs targeting hub genes were investigated using L1000 platform.

**Results:**

30 DEGs and 6 DEMIs were identified in the lung tissue of IPAH. GO and KEGG pathway analyses revealed that these DEGs were mostly enriched in antimicrobial humoral response and African trypanosomiasis, respectively. The DEMI-DEG network was conducted subsequently with 4 DEMIs (hsa-miR-34b-5p, hsa-miR-26b-5p, hsa-miR-205-5p, and hsa-miR-199a-3p) and 16 DEGs, among which 5 DEGs (AQP9, SPP1, END1, VCAM1, and SAA1) were included in the top 10 hub genes of the PPI network. Nimodipine was identified with the highest CMap connectivity score in L1000 platform.

**Conclusion:**

Our study conducted a miRNA-mRNA network and identified 4 miRNAs as well as 5 mRNAs which may play important roles in the pathogenesis of IPAH. Moreover, we provided a new insight for future therapies by predicting potential drugs targeting hub genes.

## 1. Introduction

Idiopathic pulmonary arterial hypertension (IPAH), a rare but life-threatening cardiopulmonary disease without any known associated disease or genetic cause, is characterized by progressively increased pulmonary artery pressure (PAP) and pulmonary vascular resistance (PVR) [1, 2]. New therapies such as calcium channel antagonists, endothelin receptor antagonists, and phosphodiesterase type 5 (PDE5) inhibitors have been applied to IPAH patients in the last two decades and have improved the survival rate of patients who are specifically sensitive to the drugs [3, 4]. For patients who failed to respond adequately to medical therapies, lung transplantation remained the last option. For now, IPAH is still supposed to be an incurable disease resulting in progressive loss of quality of life with a total 5-year survival rate of 51% and a 5-year survival rate of 47% despite receiving lung transplantation [5, 6]. Thus, comprehensive analyses of potential mechanisms and searching for more possible therapies in IPAH are urgently needed.

microRNA (miRNA) is a type of small, noncoding RNA, which negatively regulates the expression of targeted genes via posttranscriptional regulation [7]. Previous studies have uncovered that dysregulation of miRNAs such as miR-204 and miR-21 was associated with the pathobiology of IPAH [8–10], suggesting miRNAs maybe novel therapeutic targets. However, few studies explore the gene targets and molecular-regulated network of IPAH-relevant miRNAs, which are necessary for the development of miRNA-based treatments [11]. In this regard, systematic analyses combining transcriptomic data with miRNA data are demanded to identify essential pathways and potential drugs in IPAH.

Bioinformatic analysis has been widely used to investigate potential mechanisms in the pathology of disease since it was developed [12–14]. Recently, a few diagnostic biomarkers for IPAH have been found by application of bioinformatic analysis [15], but the miRNA expression in IPAH and their functional roles as well as mRNA targets are poorly studied.

In this study, microarray datasets of IPAH were searched and downloaded from Gene Expression Omnibus (GEO). miRNA-mRNA network was established accompanied with associated comprehensive analyses in order to better understand the mechanisms of IPAH. Additionally, potential drugs targeting hub genes were investigated to contribute to future therapies of IPAH.

## 2. Methods

### 2.1. Data Resources

The mRNA and miRNA expression profiles of IPAH patients from Gene Expression Omnibus (https://www.ncbi.nlm.nih.gov/geoprofiles/) and ArrayExpress (https://www.ebi.ac.uk/arrayexpress/) were searched. GSE113439 and GSE117261 with mRNA expression profile and GSE67597 with miRNA expression profile were identified in GEO, while no related profile was found in ArrayExpress. Subsequently, secondary PAH patients with other diseases were excluded. Finally, GSE113439 with 6 IPAH and 11 controls, GSE117261 with 32 IPAH and 25 controls, and GSE67597 with 7 IPAH and 8 controls were included in this study. Additional approval by an ethics committee was not necessary because the datasets included in the current study were downloaded from public databases.

### 2.2. Identification of DEGs and DEMIs

R-platform (http://R-project.org) and limma package [16] were used to screen the differentially expressed genes (DEGs) and differentially expressed miRNAs (DEMIs) between IPAH and healthy controls. ∣log_2_FC | >1 was considered the threshold for different expressions, and statistical difference was defined as adjusted *P* value < 0.05. Those with log_2_FC < 0 were considered as downregulated genes, while those with log_2_FC > 0 were considered as upregulated genes. The visualization of DEGs and DEMIs was realized in the volcano plot and heat map by pheatmap package in R platform. Two series (GSE113439 and GSE117261) containing mRNA expression profile were from the same platform ([HuGene-1_0-st] Affymetrix Human Gene 1.0 ST Array [transcript (gene) version]) and merged into one for analyses after batch normalization realized by sva package in R platform.

### 2.3. Functional Enrichment Analyses

R package clusterProfiler [17] was applied to conduct Gene Ontology (GO) analysis and Kyoto Encyclopedia of Genes and Genomes (KEGG) pathway enrichment analysis to identify the potential biological function of DEGs and analyze the enriched pathway of the key DEGs. Adjusted *P* value < 0.05 was considered statistically significant.

### 2.4. PPI Network Analysis

The online tool, STRING (http://string-db.org/) [18], was employed to establish protein-protein interaction (PPI) network of DEG-encoded proteins with a confidence score > 0.40. Subsequently, hub genes and core modules were selected and visualized by Cytoscape software (V3.5.1; http://cytoscape.org/) [19].

### 2.5. Prediction of miRNA-Targeted Gene and DEMI-DEG Network

To better understand the function of DEMI, miRDB (http://www.mirdb.org/), TargetScan (http://www.targetscan.org/vert_72/), TargetMiner (https://www.isical.ac.in/∼bioinfo_miu), and miRWalk (http://zmf.umm.uni-heidelberg.de/apps/zmf/mirwalk) were used to predict target genes. Predicted genes in these four databases were pooled into a solitary database, and the intersection of DEGs and the database were regarded as significantly differentially expressed target genes. Furthermore, the DEMI-DEG network was constructed by Cytoscape software.

### 2.6. Prediction of Potential Drugs for IPAH

L1000 platform (https://clue.io/) was used to explore potential drugs toward IPAH for pharmaceutical development [20]. Upregulated DEGs involved in the DEMI-DEG network were submitted to the L1000 platform for prediction of potential drugs for IPAH. Drugs with the CMap connectivity score of +90 or higher, and of -90 or lower, were considered to be potential effective drugs.

## 3. Results

### 3.1. Identification of DEGs and DEMIs

**Figure 1:**
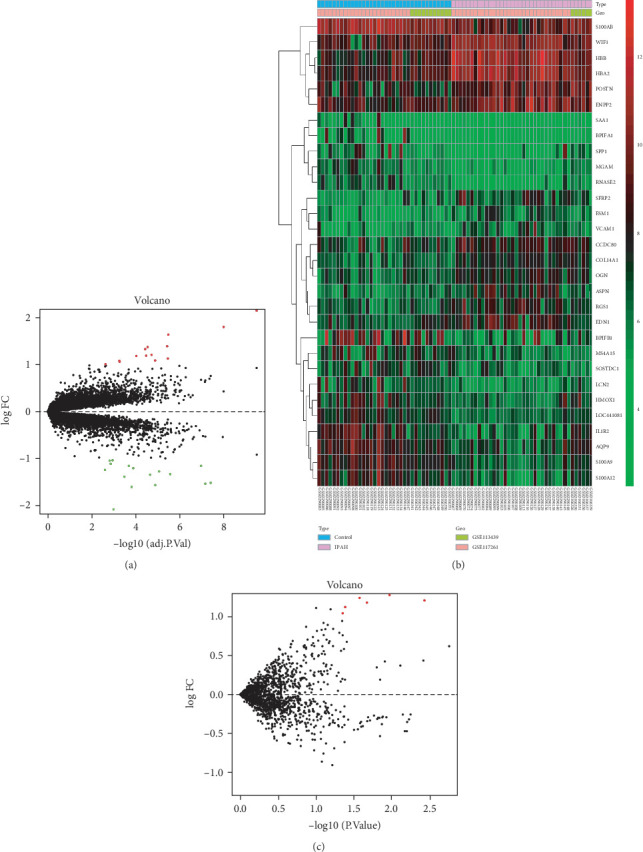
(a) Volcano plot of DEGs in IPAH. Green represents downregulated DEGs; red represents upregulated DEGs; and black represents no difference. (b) Heat map of the DEGs in IPAH compared with normal controls. Red represents greater expression and green represents less expression. (c) Volcano plot of DEMIs in IPAH. DEG: differentially expressed gene; IPAH: idiopathic pulmonary arterial hypertension; DEMI: differentially expressed miRNA.

The basic information of the datasets related to IPAH is shown in Table 1. The expression of different genes was calculated according to mapped probes, and average value was applied if multiple probes matched the same gene. Overall, 20146 genes were analyzed in GSE113439 and GSE117261. 30 DEGs were found between IPAH patients and the control group, among which 14 were upregulated and 16 were downregulated (Figure 1(a)). Furthermore, 2006 miRNAs were analyzed and 6 DEMIs were found, indicating these 6 DEMIs may participate in the pathogenesis of IPAH (Figure 1(b)). Details of DEGs and DEMIs between IPAH patients and the control are showed in Supplement Tables 1 and 2, respectively.

### 3.2. Functional Enrichment Analyses

**Figure 2:**
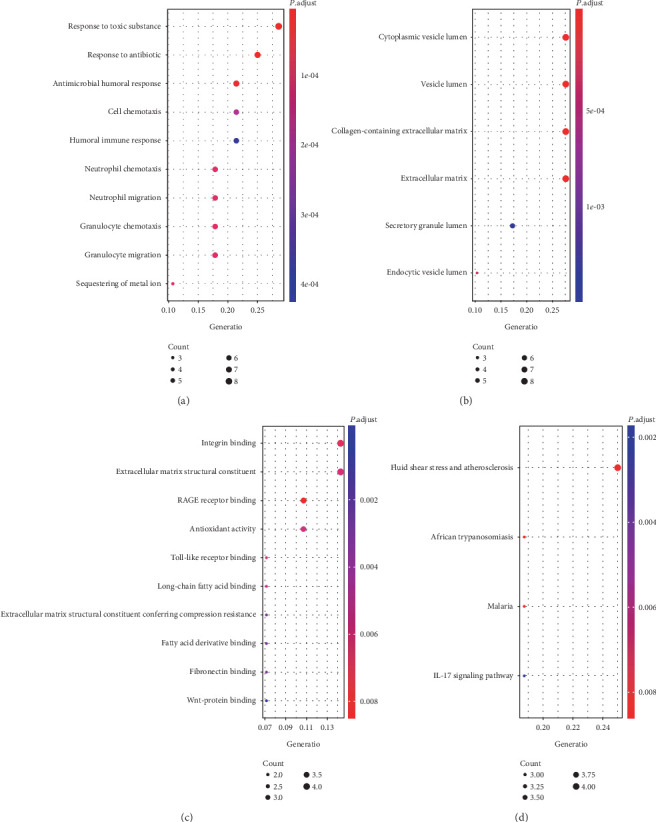
The top 10 enriched Gene Ontology terms in biological process (a), cellular component (b), molecular function (c) and enriched Kyoto Encyclopedia of Genes and Genomes pathway (d) of DEGs. DEG: differentially expressed gene.

Subsequently, functional enrichment analyses were performed to reveal the function of DEGs and the results are shown in Figure 2.

Go enrichment analysis revealed that DEG-related biological processes (BP) were mostly enriched in antimicrobial humoral response (*P* value < 0.0001), neutrophil chemotaxis (*P* value < 0.0001), and neutrophil migration (*P* value < 0.0001), while cellular components (CC) were mainly enriched in cytoplasmic vesicle lumen (*P* value < 0.0001), vesicle lumen (*P* value < 0.0001), and collagen-containing extracellular matrix (*P* value < 0.0001). In addition, molecular function (MF) analyses suggested that DEGs were involved in RAGE receptor binding (*P* value = 0.0016), integrin binding (*P* value = 0.0159) and Toll-like receptor binding (*P* value = 0.0029).

To investigate the crucial pathways of these DEGs, KEGG pathways analysis was performed and the significant pathways are shown in Figure 2. The DEGs were enriched in the pathway of African trypanosomiasis (*P* value = 0.0020), malaria (*P* value = 0.0020), fluid shear stress and atherosclerosis (*P* value = 0.0020), and IL-17 signaling pathway (*P* value = 0.0085).

### 3.3. PPI Network Analysis

**Figure 3:**
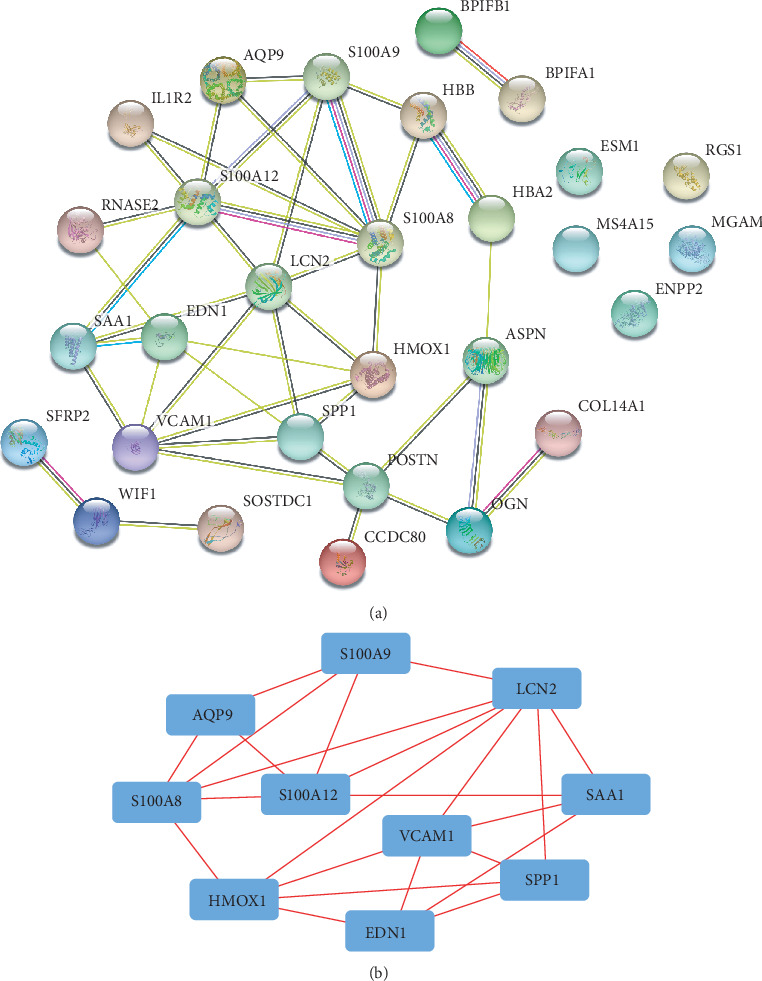
Protein-protein interaction network analysis (a) and hub genes (b) identified by Cytoscape.

Using the STRING database, we performed PPI network analysis for DEGs. As we have shown in Figure 3, 24 nodes were mainly identified. AQP9, EDN1, S100A12, S100A8, S100A9, SPP1, HMOX1, VCAM1, LCN2, and SAA1 were the top 10 hub genes.

### 3.4. Prediction of miRNA-Targeted Gene and DEMI-DEG Network

**Figure 4:**
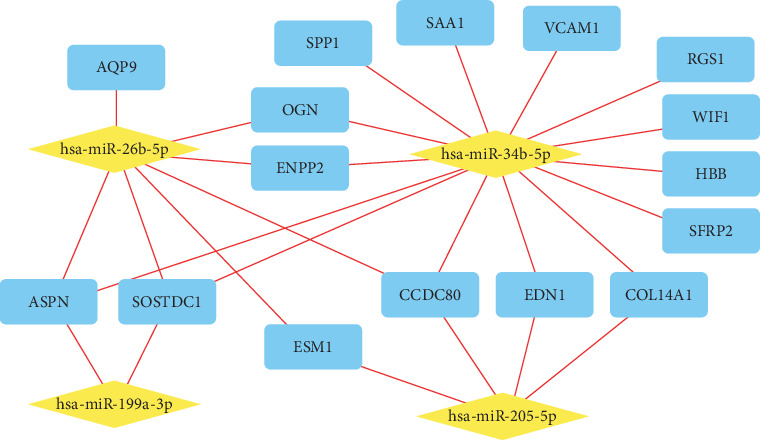
DEMI-DEG network. Rhombus represents miRNA while rectangle represents mRNA. DEG: differentially expressed gene; DEMI: differentially expressed miRNA.

In order to further identify important miRNA-mRNA-regulated axes in IPAH, four databases (miRDB, TargetScan, TargetMiner, and miRWalk) were employed to predict the target genes of DEMIs. 9095 genes were predicted as target genes of 6 DEMIs. GO and KEGG analyses of those genes were performed with results shown in Supplement Figure 1. Among the target genes of DEMIs, 16 were intersected with DEGs (HBB, COL14A1, WIF1, OGN, RGS1, ASPN, ESM1, SFRP2, ENPP2, EDN1, VCAM1, CCDC80, AQP9, SAA1, SOSTDC1, and SPP1), which were used to construct the DEMI-DEG network (Figure 4). The network consisted of 4 DEMIs, 16 predicted target DEGs, and 27 edges, while hsa-miR-99a-5p and hsa-miR-30a-5p were connected with no predicted target DEGs and were excluded from the network. hsa-miR-34b-5p was related to most of the predicted target DEGs (14 edges), followed by hsa-miR-26b-5p (7 edges), hsa-miR-205-5p (4 edges), and hsa-miR-199a-3p (2 edges). In the other hand, ASPN, CCDC80, and SOSTDC1 were connected with 3 DEMIs, respectively. Details of DEGs and DEMIs in the DEMI-DEG network are showed in Tables 2 and 3, respectively.

### 3.5. Prediction of Potential Drugs for IPAH

The 12 upregulated DEGs in the DEMI-DEG network were uploaded to the L1000 platform to search for potential drugs, and the top 20 ranked by CMap connectivity score are listed in Table 4.

## 4. Discussion

Though new therapies have been developed in recent years, IPAH remains a severe disease with continuous suffering to patients and society. Therefore, better understanding of potential mechanisms in the pathogenesis of IPAH may shed a light for future studies.

In the present study, 30 DEGs, mostly related to immune and inflammatory response such as neutrophil chemotaxis and migration, integrin binding and Toll-like receptor binding, and significantly enriched in inflammation pathways like IL-17 signaling pathway, and 6 DEMIs were found in IPAH. The DEMI-DEG network was conducted subsequently with 4 DEMIs (hsa-miR-34b-5p, hsa-miR-26b-5p, hsa-miR-205-5p, and hsa-miR-199a-3p) and 16 DEGs. We found that 5 DEGs (AQP9, SPP1, END1, VCAM1, and SAA1) were included in the top 10 hub genes of the PPI network, indicating those factors may play important roles in the pathogenesis of IPAH. Finally, drugs including nimodipine were identified. To our knowledge, this is the first study that conducted the network of mRNA and miRNA and predicted potential drugs in IPAH, which provides a foundation for further research and development of therapy.

Previous studies have revealed the important role of the miRNAs and mRNAs that is identified in our DEMI-DEG network in the pathogenesis of IPAH. Wu et al. identified dysregulated miRNAs in IPAH via miRNA profiling and qRT-PCR. miR-26b-5p and miR-199a-3p were detected as two of the top 5 most significantly increased miRNAs targeting major PAH related pathways including Wnt/*β*-catenin in end-stage IPAH [21]. In pulmonary vascular smooth muscle cells (PASMCs), miR-205-5p was reported to suppress cell proliferation by targeting MICAL2-mediated Erk1/2 signaling, which may be regarded as a therapeutic target in PAH [22]. For mRNA, 5 mRNAs of the top 10 hub genes in the PPI network were included in our DEMI-DEG network. Among them, endothelin-1 (EDN1) is a potent vasoconstrictor and its receptors are therapeutic targets in the treatment of PAH [23, 24]. It was able to promote contraction via increasing intracellular Ca^2+^ and increasing PASMC proliferation and migration, leading to vascular remodeling, which is a key mechanism underlying PAH [25–27]. Vascular cell adhesion molecule 1 (VCAM1), an adhesion molecule mediating leukocyte transmigration and increasing tissue inflammation [28], was found to increase in both patients and animal models of IPAH [29, 30]. Aquaporin (AQP), a family of water-selective membrane channels promoting endothelial cell migration and angiogenesis [31], was found to be overexpressed in PAH patients [32, 33], indicating its potential role in the treatment of PAH. Secreted phosphoprotein 1 (SPP1, also known as osteopontin or OPN), a key mediator secreted by SMCs, contributes to the genesis and progression of pulmonary hypertension by enhancing PVSMC proliferation [34–36]. It is upregulated in the lung tissues of patients, and the SPP1 expression level is associated with the severity of PAH [37–39]. These results demonstrated that SPP1 may be a prognostic marker as well as a therapeutic target in PAH.

However, some of the factors in our DEMI-DEG network were poorly studied. The role of hsa-miR-34b-5p, which was connected with most of the predicted target DEGs, has not been studied in IPAH before. miR-34b was known to affect cell proliferation and adhesion-independent growth in several types of cancer [40, 41]. Lin et al. previously discovered DNA methylation of miR-34b-regulated vascular calcification by targeting Notch1 [42]. Furthermore, serum amyloid A1 (SAA1), an acute phase protein, is primarily synthesized in the liver and highly expressed when inflammatory response occurs [43]. The elevated SAA1 concentration was associated with cardiovascular risk [44], carotid intima media thickness [45], and smooth muscle cell homeostasis [46] according to previous researches. Whether and how these factors contribute to IPAH need to be investigated in future studies.

The calcium channel blocker is an effective treatment for IPAH patients with a positive vasodilator response, which dramatically improves the survival of those patients and has been widely applied recent years [47, 48]. Nimodipine, a kind of calcium channel blocker, was identified as the top of the potential drugs in our research. However, other calcium channel blockers were not predicted with a high rank, so whether nimodipine has a clinical benefit cannot be determined. Furthermore, other drugs in the prediction list, which were hardly researched in IPAH before, may serve as potential directions for future development of therapies in IPAH.

There are some limitations in our study. Firstly, only two mRNA expression profiles and one miRNA expression profile were found in GEO; the relatively small number of samples may make the results less convincing. Secondly, since the expression profiles of miRNA and mRNA were obtained from different datasets in this study, coexpression analysis cannot be performed currently, which prevents us from obtaining a more accurate miRNA-mRNA regulation network. In addition, PCR or WB should be done to further confirm our results, and the relationship between miRNAs and mRNAs in our DEMI-DEG network should be validated via *in vivo* or *in vitro* experiments.

## 5. Conclusion

Our study initially conducted a miRNA-mRNA network including 4 miRNAs and 5 mRNAs to systematically analyze the pathogenesis of IPAH and provided a new insight for future therapies by predicting potential drugs acting with hub genes.

## Figures and Tables

**Table 1:** Details of datasets related to IPAH patients.

	GEO ID	Platform	Organism	Experiment type	Samples (case vs. control)	Country	Year
mRNA	GSE117261	GPL6244	Homo sapiens	Expression profiling by array	32 vs. 25	United States of America	2018
	GSE113439	GPL6244	Homo sapiens	Expression profiling by array	6 vs. 11	Canada	2018
miRNA	GSE67597	GPL18402	Homo sapiens	Noncoding RNA profiling by array	7 vs. 8	United States of America	2015

IPAH: idiopathic pulmonary arterial hypertension; GEO; Gene Expression Omnibus.

**Table 2:** Details of the 16 DEGs included in DEMI-DEG network.

Gene	Ensembl ID	Log_2_FC	Adjusted *P* value
HBB	ENSG00000244734	2.1829	3.26*E*-10
COL14A1	ENSG00000187955	1.1296	3.55*E*-06
WIF1	ENSG00000156076	1.3922	3.82*E*-06
OGN	ENSG00000106809	1.0880	1.34*E*-05
RGS1	ENSG00000090104	1.2087	1.95*E*-05
AQP9	ENSG00000103569	-1.3423	2.29*E*-05
ASPN	ENSG00000106819	1.3752	2.96*E*-05
ESM1	ENSG00000164283	1.1911	3.45*E*-05
SFRP2	ENSG00000145423	1.3301	3.83*E*-05
ENPP2	ENSG00000136960	1.1842	9.72*E*-05
SAA1	ENSG00000173432	-1.1547	0.0002
EDN1	ENSG00000078401	1.0631	0.0006
VCAM1	ENSG00000162692	1.0820	0.0006
SOSTDC1	ENSG00000171243	-1.1114	0.0014
CCDC80	ENSG00000091986	1.0089	0.0024
SPP1	ENSG00000118785	-1.2404	0.0026

DEG: differentially expressed gene; DEMI: differentially expressed miRNA; FC: fold change.

**Table 3:** Details of the 4 DEMIs included in the DEMI-DEG network.

Gene	miRBase	Log_2_FC	*P* value
hsa-miR-205-5p	MIMAT0000266	1.2088	0.0038
hsa-miR-199a-3p	MIMAT0000232	1.2975	0.0109
hsa-miR-34b-5p	MIMAT0000685	1.2421	0.0270
hsa-miR-26b-5p	MIMAT0000083	1.1240	0.0418

DEG: differentially expressed gene; DEMI: differentially expressed miRNA; FC: fold change.

**Table 4:** The top 10 and bottom 10 chemical compounds identified by L1000 platform.

Name	CMap connectivity score	Description
Nimodipine	99.89	Calcium channel blocker
GW-501516	99.82	PPAR receptor agonist
Icilin	99.82	TRPV agonist
Fexofenadine	99.82	Histamine receptor antagonist
Modafinil	99.79	Adrenergic receptor agonist
Guanabenz	99.79	Alpha-2 selective adrenergic agonist
Mianserin	99.75	Serotonin receptor antagonist
CGP-53353	99.75	EGFR inhibitor
TUL-XXI039	99.72	Serine/threonine kinase inhibitor
Diphenoxylate	99.68	Opioid receptor agonist
Huperzine-a	-99.46	Acetylcholinesterase inhibitor
Perindopril	-99.47	ACE inhibitor
Solanine	-99.47	Acetylcholinesterase inhibitor
Phenothiazine	-99.51	Dopamine receptor antagonist
Tyrphostin-AG-1295	-99.68	PDGFR receptor inhibitor
Y-27152	-99.72	Potassium channel activator
Lenalidomide	-99.79	Antineoplastic
Androstenedione	-99.89	Cytochrome P450 inhibitor
Repaglinide	-99.89	Insulin secretagogue
Neurodazine	-99.89	Neurogenesis of nonpluripotent C2C12 myoblast inducer

## Data Availability

All data generated or analysed during this study are included in this published article [and its supplementary information files].

## Abbreviations

IPAH: Idiopathic pulmonary arterial hypertension
miRNA: microRNA
DEGs: Differentially expressed genes
DEMIs: Differentially expressed miRNAs
GEO: Gene Expression Omnibus
GO: Gene Ontology
KEGG: Kyoto Encyclopedia of Genes and Genomes
PPI: Protein-protein interaction
BP: Biological processes
CC: Cellular components
MF: Molecular function
PAP: Pulmonary artery pressure
PVR: Pulmonary vascular resistance
PDE5: Phosphodiesterase type 5
PASMC: Pulmonary vascular smooth muscle cell
EDN1: Endothelin-1
VCAM1: Vascular cell adhesion molecule 1
AQP9: Aquaporin 9
SPP1: Secreted phosphoprotein 1
SAA1: Serum amyloid A1.

## Supplementary Materials

Supplementary Materials Supplement Table 1: the 30 DEGs in the lung tissue of patients with IPAH. Supplement Table 2: the 6 DEMIs in the lung tissue of patients with IPAH. Supplement Figure 1: the top 10 enriched Gene Ontology terms in biological process (A), cellular component (B), molecular function (C), and enriched Kyoto Encyclopedia of Genes and Genomes pathway (D) of the targets of DEMIs. DEMIs: differential expressed miRNAs.
