# Supplementary material for: Comprehensive Analyses of miRNA-mRNA Network and Potential Drugs in Idiopathic Pulmonary Arterial Hypertension
Source: Biomed Res Int. 2020 Jul 3;2020:5156304. doi: 10.1155/2020/5156304 (PMC7355352; doi:10.1155/2020/5156304)
Supplement: Supplementary Materials — Supplement Table 1: the 30 DEGs in the lung tissue of patients with IPAH. Supplement Table 2: the 6 DEMIs in the lung tissue of patients with IPAH. Supplement Figure 1: the top 10 enriched Gene Ontology terms in biological process (A), cellular component (B), molecular function (C), and enriched Kyoto Encyclopedia of Genes and Genomes pathway (D) of the targets of DEMIs. DEMIs: differential expressed miRNAs. [file 5156304.f1.docx]

**Supplement table 1. The 30 DEGs in the lung tissue of patients with IPAH.**

| Gene | Log_2_FC | adjust P value |
| --- | --- | --- |
| HBB | 2.1829 | 3.26E-10 |
| HBA2 | 1.8026 | 1.03E-08 |
| RNASE2 | -1.5159 | 4.00E-08 |
| S100A9 | -1.5354 | 7.01E-08 |
| LOC441081 | -1.1537 | 1.10E-07 |
| S100A8 | -1.3316 | 2.77E-06 |
| POSTN | 1.6389 | 3.39E-06 |
| COL14A1 | 1.1296 | 3.55E-06 |
| WIF1 | 1.3922 | 3.82E-06 |
| MGAM | -1.2719 | 8.89E-06 |
| S100A12 | -1.5648 | 1.32E-05 |
| OGN | 1.0880 | 1.34E-05 |
| RGS1 | 1.2087 | 1.95E-05 |
| AQP9 | -1.3423 | 2.29E-05 |
| ASPN | 1.3752 | 2.96E-05 |
| ESM1 | 1.1911 | 3.45E-05 |
| SFRP2 | 1.3301 | 3.83E-05 |
| ENPP2 | 1.1842 | 9.72E-05 |
| LCN2 | -1.2038 | 0.0001 |
| IL1R2 | -1.6033 | 0.0002 |
| SAA1 | -1.1547 | 0.0002 |
| BPIFA1 | -1.3853 | 0.0003 |
| EDN1 | 1.0631 | 0.0006 |
| VCAM1 | 1.0820 | 0.0006 |
| BPIFB1 | -2.0815 | 0.0010 |
| HMOX1 | -1.0357 | 0.0012 |
| SOSTDC1 | -1.1114 | 0.0014 |
| MS4A15 | -1.0436 | 0.0016 |
| CCDC80 | 1.0089 | 0.0024 |
| SPP1 | -1.2404 | 0.0026 |

DEG, differentially expressed gene; IPAH, idiopathic pulmonary arterial hypertension; FC, fold change.

**Supplement table 2. The 6 DEMIs in the lung tissue of patients with IPAH.**

| Gene | log_2_FC | P value |
| --- | --- | --- |
| hsa-miR-205-5p | 1.2088 | 0.0038 |
| hsa-miR-199a-3p | 1.2975 | 0.0109 |
| hsa-miR-99a-5p | 1.1813 | 0.0216 |
| hsa-miR-34b-5p | 1.2421 | 0.0270 |
| hsa-miR-26b-5p | 1.1240 | 0.0418 |
| hsa-miR-30a-5p | 1.0444 | 0.0451 |

DEMI, differentially expressed miRNA; IPAH, idiopathic pulmonary arterial hypertension; FC, fold change.


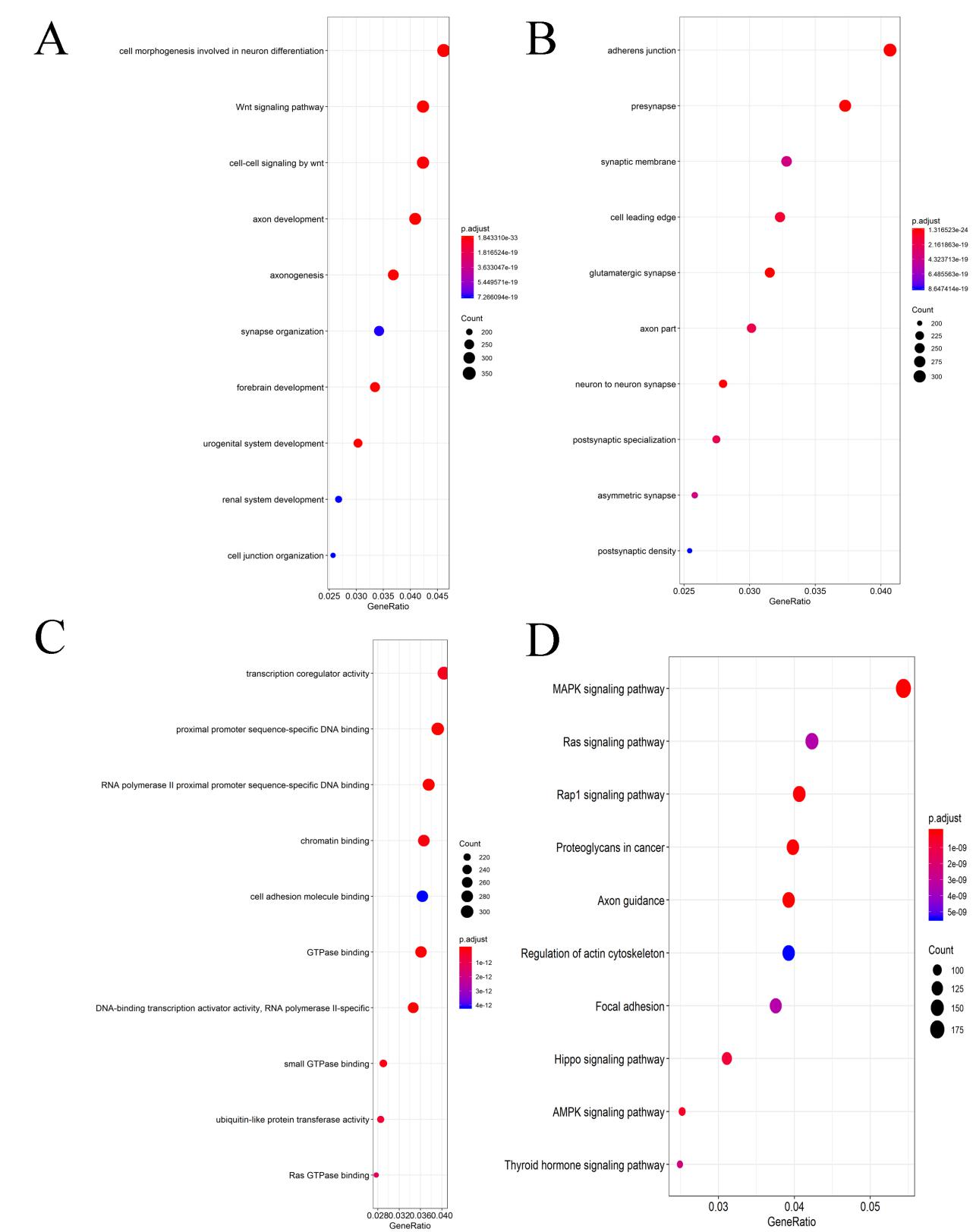
Supplement Figure 1. The top 10 enriched Gene Ontology terms in biological process (A), cellular component (B), molecular function (C) and enriched Kyoto Encyclopedia of Genes and Genomes pathway (D) of the targets of DEMIs.

DEMIs，differentially expressed miRNAs
